# Tigecycline pharmacokinetics in critically ill patients receiving extracorporeal membrane oxygenation: a prospective multicentre pilot study

**DOI:** 10.1186/s12871-026-04114-1

**Published:** 2026-08-06

**Authors:** Rawan Alraish, Afrah Alkazemi, Kamal M. Matar, Sara F. Buabbas, Abdulaziz Al-Mutawa, Abdulrahman Al-fares

**Affiliations:** 1 Department of Anaesthesiology and Intensive Care Medicine, Charité – Universitätsmedizin Berlin, Augustenburger Platz 1, 13353 Berlin, Germany; 2 Department of Pharmacy Practice, College of Pharmacy, Kuwait University, Safat, PO Box 24923, Kuwait City, 13110 Kuwait; 3 Department of Pharmacology and Therapeutics, College of Pharmacy, Kuwait University, Kuwait City, Kuwait; 4 Department of Anaesthesia, Critical Care Medicine and Pain Medicine, Jaber Al-Ahmad Hospital, Ministry of Health, Kuwait City, Kuwait; 5 Department of Anaesthesia, Critical Care Medicine and Pain Medicine, Al-Amiri Hospital, Ministry of Health, Kuwait City, Kuwait; 6 Kuwait Extracorporeal Life Support Program, Al-Amiri Hospital, Ministry of Health, Kuwait City, Kuwait

**Keywords:** Tigecycline, Pharmacokinetics, ECMO, Critically ill, Therapeutic drug monitoring

## Abstract

**Background:**

Tigecycline is frequently used to treat multidrug-resistant infections in critically ill patients. However, its pharmacokinetics in patients receiving extracorporeal membrane oxygenation (ECMO) are poorly defined, raising concerns about the adequacy of standard dosing in this population.

**Methods:**

This prospective, multicentre pilot study included adult ICU patients receiving venovenous (VV) or venoarterial (VA) ECMO and standard tigecycline dosing. Serial plasma samples were collected at steady state to determine tigecycline concentrations using liquid chromatography–tandem mass spectrometry. Pharmacokinetic parameters, including maximum plasma concentration (C_max_), trough plasma concentration (C_min_), area under the concentration–time curve (AUC), and systemic clearance (CL), were calculated. Microbiological samples were obtained before therapy initiation. Pharmacokinetic parameters were compared between ECMO configurations and correlations with organ function markers were assessed using Spearman analysis.

**Results:**

Fifteen patients were included (13 VV-ECMO, 2 VA-ECMO). Marked interindividual variability in tigecycline exposure was observed. Median C_max_ was 0.50 µg/mL, with consistently low trough concentrations. Plasma levels generally exceeded EUCAST MIC breakpoints for Gram-positive pathogens but were lower for Gram-negative organisms. Patients receiving VA-ECMO exhibited numerically higher tigecycline exposure than those on VV-ECMO. Moderate, non-significant positive correlations were observed between total bilirubin levels and tigecycline exposure.

**Conclusion:**

Tigecycline pharmacokinetics in ECMO-supported critically ill patients show high variability, with higher exposure in VA-ECMO than in VV-ECMO. Standard dosing may be insufficient, supporting therapeutic drug monitoring and further studies to inform individualized dosing strategies in this complex population.

## Introduction

Extracorporeal membrane oxygenation (ECMO) is increasingly implemented in intensive care units (ICUs) worldwide to support patients with life-threatening respiratory or cardiac failure unresponsive to conventional therapy. Recent data from the Extracorporeal Life Support Organization (ELSO) indicate a continuous year-over-year rise in ECMO utilization across diverse patient populations and age groups [1]. While ECMO has revolutionized the management of severe cardiac and respiratory failure, its implementation introduces profound physiologic alterations that can significantly affect drug pharmacokinetics (PK) and pharmacodynamics (PD). These alterations are driven by factors such as increased volume of distribution due to hemodilution, adsorption or sequestration of drugs within ECMO circuit components including the oxygenator and tubing, and changes in organ perfusion and function that may alter drug metabolism and elimination [2–4]. Consequently, subtherapeutic antimicrobial exposure may result in treatment failure and resistance development, while conversely excessive concentrations can increase toxicity risk in this critically ill and often multi-organ impaired patient group [5].

Tigecycline (TGC), a glycylcycline antibiotic structurally related to minocycline, is approved for the treatment of complicated intra-abdominal infections, complicated skin and soft tissue infections, and community-acquired bacterial pneumonia [6]. It demonstrates broad-spectrum activity, including efficacy against multidrug-resistant organisms such as methicillin-resistant *Staphylococcus aureus* (MRSA), vancomycin-resistant *Enterococcus* spp., extended-spectrum β-lactamase–producing Enterobacterales, and *Acinetobacter baumannii* [7, 8]. The pharmacokinetic profile of TGC is characterized by an extensive volume of distribution (7–10 L/kg), moderate plasma protein binding (~ 70–80%), and predominant hepatic elimination via glucuronidation and biliary excretion, with minimal renal clearance [9, 10]. Current dosing recommendations suggest dose adjustments only in patients with severe hepatic impairment (Child-Pugh C), while no modifications are required in renal impairment [11].

In critically ill patients, population pharmacokinetic studies have identified hepatic function markers such as total bilirubin and gamma-glutamyl transferase as significant covariates influencing TGC clearance and intercompartmental distribution [12]. Notably, König et al. demonstrated that reduced liver function, assessed by the LiMAx test, correlated with increased TGC C_max_ in ICU patients with liver impairment [13]. However, despite these insights, there remains substantial interindividual variability in TGC exposure among critically ill patients, which may be further exacerbated in those supported with ECMO.

Evidence regarding TGC pharmacokinetics during ECMO is limited to a few reports. Veinstein et al. published a 2012 case report describing TGC PK in a single ECMO patient, suggesting minimal circuit-related alterations [14]. Similarly, Zhang et al. conducted an ex vivo circuit study that found no significant sequestration of TGC within ECMO circuits [15]. Nonetheless, these findings lack validation in larger multicenter in vivo cohorts, and recent reviews emphasize the persistent gap in robust dosing recommendations for antimicrobials in ECMO settings, highlighting the potential role of therapeutic drug monitoring (TDM) to guide therapy [16, 17].

Therefore, to address this critical knowledge gap, we conducted a prospective multicentre pilot pharmacokinetic study of TGC in adult patients undergoing ECMO. The primary objective was to characterize TGC exposure over a dosing interval in ECMO-supported patients. Secondary objectives included comparing pharmacokinetic parameters between venovenous (VV-ECMO) and venoarterial (VA-ECMO) configurations, as well as evaluating whether standard dosing achieves adequate systemic exposure relative to EUCAST minimum inhibitory concentration (MIC) breakpoints for common Gram-positive and Gram-negative pathogens.

## Methods

### Study design and population

This was a prospective multicenter observational pilot study conducted at Al-Amiri Hospital and Jaber Al-Ahmad Al-Sabah Hospital in Kuwait. Ethical approval was obtained from the Kuwait Ministry of Health by local protocols (2022/1994). Adult critically ill patients receiving either venovenous (VV-ECMO) or venoarterial (VA-ECMO) support in the intensive care unit (ICU) were screened for eligibility. Inclusion criteria were: male or female patients ≥ 18 years of age; written informed consent obtained from the patients or their legal representative; and the ability of the patients to comply with the requirements of the study. Exclusion criteria included receipt of TGC prior to ECMO initiation; known allergy to TGC or concomitant medications that interact with TGC metabolism; ongoing massive blood transfusion requirement (> 50% blood volume transfused in the previous 8 h); therapeutic plasma exchange within the preceding 24 h; pregnancy; or any condition or abnormality that, in the opinion of the investigator, would compromise patient safety or data quality.

All patients received the standard FDA-approved TGC dosing regimen: a 100 mg intravenous loading dose infused over 30 min, followed by maintenance doses of 50 mg/30min every 12 h. Microbiological samples were collected prior to TGC initiation to identify the causative pathogens. According to local guidelines, patients with septic shock or infections caused by TGC-resistant pathogens received combination therapy with other broad-spectrum antibiotics. Pharmacokinetic sampling was performed after at least 36 h of TGC therapy following repeated maintenance dosing, consistent with our previously published TGC pharmacokinetic methodology [17] and other studies evaluating TGC pharmacokinetics after repeated dosing in critically ill patients [18].

### Pharmacokinetic sampling and clinical data collection

On the pharmacokinetic sampling day, blood samples were collected at 0.3, 2, 5, 8, and 11.5 h after the end of TGC infusion. Plasma was separated by centrifugation and stored at -80 °C at each participating site. The frozen plasma samples were shipped on dry ice to the central laboratory at the College of Pharmacy, University of Kuwait, for analysis. Routine laboratory parameters were collected on the same day, including static liver function tests [aspartate aminotransferase (AST), alanine aminotransferase (ALT), gamma-glutamyl transferase (GGT), pseudocholinesterase (PCHE), glutamate dehydrogenase (GLDH), alkaline phosphatase (ALP), total bilirubin], lactate, platelet count, and international normalized ratio (INR). Baseline demographic and clinical data were also recorded, including age, gender, weight, body mass index (BMI), relevant past medical history, APACHE II score on ICU admission, and SOFA score on the day of pharmacokinetic sampling. Microbiological results, ECMO configuration and modality, and antimicrobial dosing data were collected as part of the study dataset and were determined by the treating clinicians according to local protocols. All subsequent patient management decisions, including the addition of other medications and the use of renal replacement therapy, were at the discretion of the treating team. The presence and modality of renal replacement therapy at the time of sampling were documented.

### TGC plasma samples preparation and analysis

TGC plasma samples were quantified using a previously reported liquid chromatographic–tandem mass spectrometric (LC-MS/MS) method [19]. The method was optimized to ensure simplicity, robustness, accuracy, and rapid turnaround. Stock solutions of TGC and tigecycline-d9 (1 mg/mL in methanol) were prepared in light-protected tubes. Calibration standards ranged from 0.25 to 10 µg/mL, and quality control (QC) samples were prepared at 0.5, 4, and 9 µg/mL by spiking drug-free plasma with TGC standard solution. Samples were stored at − 80 °C pending analysis.

Plasma samples were thawed at room temperature and subjected to protein precipitation with pre-cooled acetonitrile. Specifically, 20 µL of plasma sample was mixed with 20 µL of internal standard, followed by 100 µL of acetonitrile. After vortex-mixing and centrifugation at 14,000 rpm for 15 min, 100 µL of the supernatant was transferred to autosampler vials. A 10 µL aliquot was injected into the LC-MS/MS system. The method showed linearity over a range of 0.05–10 µg/mL. Intra- and inter-batch precision values were within 7.2%, and accuracy ranged from 93.4% to 101.8%. Total analysis time per sample was approximately 2 min.

### Pharmacokinetic analysis

Plasma TGC concentrations were analyzed using non-compartmental methods (NCA). Samples were collected at steady state over one complete dosing interval following the maintenance dose. The area under the plasma concentration–time curve during one dosing interval at steady state (AUC₀–₁₂,ss) was calculated using the linear-up/log-down trapezoidal method in Microsoft Excel (Microsoft Corp., Redmond, WA, USA).

Because TGC was administered intravenously, Clearance (CL) was calculated as:

$$\:C\mathrm{L}=\frac{D}{AU{C}_{0-12}}$$

where D represents the TGC maintenance dose administered during one dosing interval (50 mg), and AUC₀₋₁₂ represents the area under the concentration-time curve over the 12-hour dosing interval at steady state. Pharmacokinetic parameters were summarized as median [interquartile range].

To assess the adequacy of TGC exposure, plasma concentration–time profiles were compared with EUCAST MIC breakpoints for relevant Gram-positive (0.25 µg/mL) and Gram-negative (0.5 µg/mL) pathogens and were used solely as a descriptive reference for observed plasma exposure [20].

### Statistical analysis

All statistical analyses were conducted using SPSS Statistics version 22 (IBM Corp., Armonk, NY, USA). Continuous variables were summarized as medians with interquartile ranges (IQR) because of their non-normal distribution, whereas categorical variables were presented as frequencies and percentages. Normality of continuous variables was assessed using the Shapiro–Wilk test and visual inspection of histograms. Pharmacokinetic parameters were summarized descriptively according to ECMO configuration (VV-ECMO and VA-ECMO). Spearman’s rank correlation coefficients were calculated to assess the associations between pharmacokinetic parameters (C_max_, C_min_, AUC, and CL) and markers of organ function. A two-sided p-value < 0.05 was considered statistically significant. Owing to the limited number of patients receiving VA-ECMO (*n* = 2), comparisons between ECMO configurations were considered exploratory and are presented descriptively. Missing data were handled using pairwise deletion.

## Results

### Demographic and clinical characteristics

Fifteen patients were enrolled in the study, with 13 (87%) receiving venovenous ECMO (VV-ECMO) and 2 (13%) receiving venoarterial ECMO (VA-ECMO). The primary indications for ECMO support were acute respiratory distress syndrome and cardiogenic shock. TGC therapy was initiated following ECMO cannulation in all patients, with a median therapy duration of 5 days (IQR: 4–5 days). The median age of the cohort was 41 years (IQR: 34–45), and the median body mass index (BMI) was 28.1 kg/m² (IQR: 26–31). The median APACHE II score on admission was 17 (IQR: 12–20), while the median SOFA score on the day of pharmacokinetic sampling was 7 (IQR: 4–13). Table 1 summarizes the demographic data and clinical characteristics of the study group.

**Table 1:** Baseline characteristics

Variable	All patients ( *n* = 15)
Age (years)	41 (34–45)
Gender (male/female)	7/8
Weight (kg)	73.5 ( 67.5–89)
BMI (kg/m²)	28.1 (26–31)
APACHE II score	17 (12–20)
SOFA score (on sampling day)	7 (4–13)
Days of therapy (days)	5 (4–5)
ECMO configuration n (%)	
VV-ECMO	13 (87%)
VA-ECMO	2 (13%)
ECMO indication n (%)	
ARDS	13 (87%)
Cardiogenic shock	2 (13%)
ECMO flow rate (L/min)	4 (3.4–4.5)
ECMO sweep gas (L/min)	2 (0.5-3)
Renal replacement therapy n (%)	3 (20%)
Total bilirubin (µmol/L)	18 (12–26)
Creatinine (µmol/L)	62 (58–160)
Microbiological results, n (%)	
Enterococcus faecalis	1 (7%)
MRSA	1 (7%)
Staph capitis	1 (7%)
Streptococcus pyogenes	1 (7%)
MDRGN	5 (33%)

Values presented as median and interquartile range

*Abbreviations*: *BMI* body mass index, *APACHE II* Acute Physiology and Chronic Health Evaluation II, *SOFA* Sequential Organ Failure Assessment, *VV-ECMO* venovenous ECMO, *VA-ECMO* venoarterial ECMO, *ARDS* acute respiratory distress syndrome, *AST* aspartate aminotransferase, *ALT* alanine aminotransferase, *MDRGN* multidrug-resistant gram-negative bacteria

### Pharmacokinetics of TGC

**Fig. 1:**
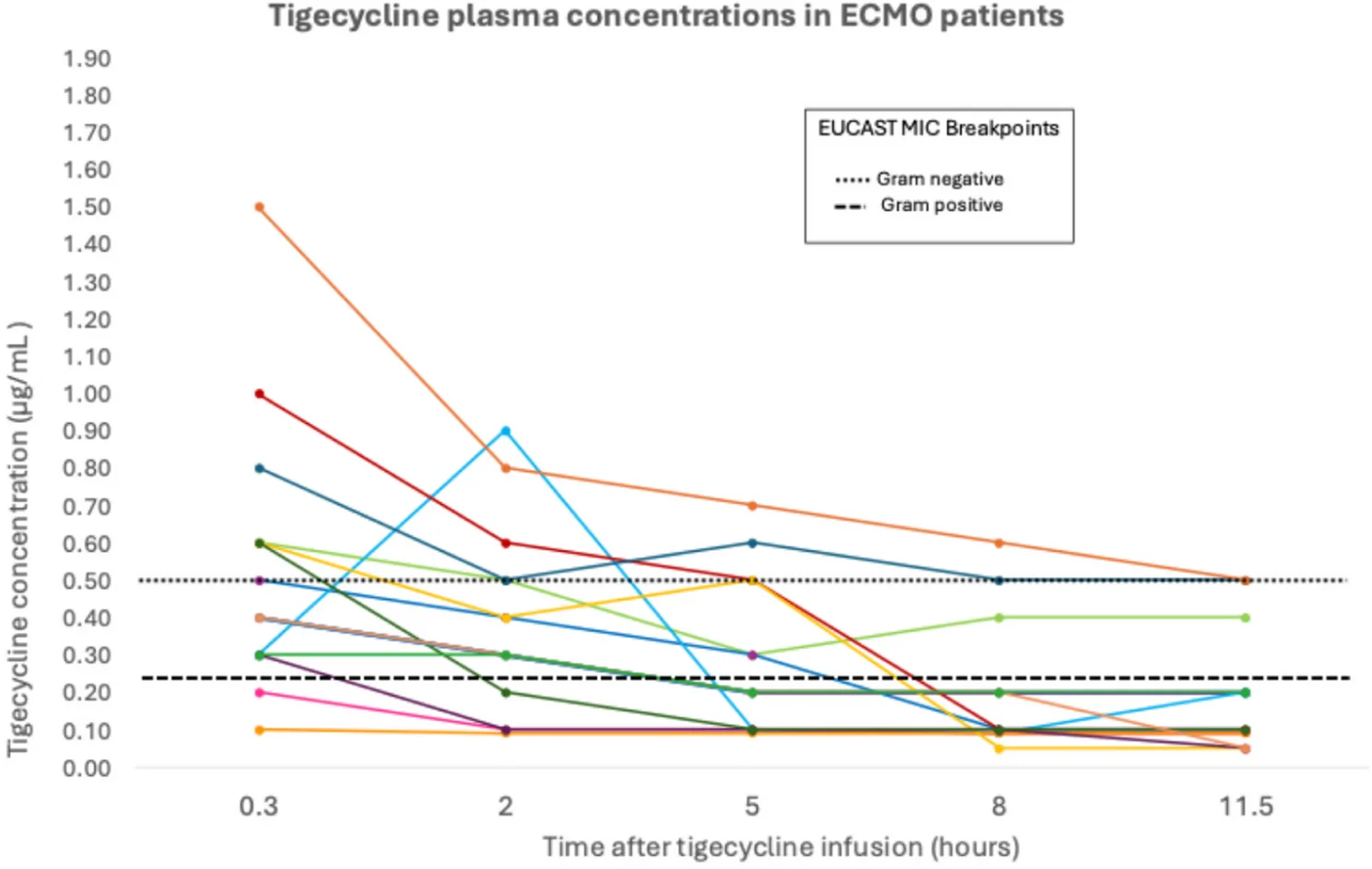
Tigecycline plasma concentrations in ECMO patients

A total of 74 plasma samples were obtained from 15 patients. The mean TGC plasma concentrations over time are shown in Fig. 1. Overall, the concentration-time profiles showed that peak levels **(**mean C_max_ = 0.573 µg/mL**)** occurred 20 min after TGC administration, followed by a decline to a trough level **(**mean C_min_ = 0.181 µg/mL) measured shortly before the next dose. Median concentrations at predefined sampling timepoints were 0.40 µg/mL (IQR: 0.30–0.6) at 20 min, 0.30 µg/mL (IQR: 0.25–0.5) at 2 h, 0.20 µg/mL (IQR: 0.1–0.4) at 5 h, 0.10 µg/mL (IQR: 0.09–0.2) at 8 h, and 0.10 µg/mL (IQR: 0.09–0.2) at 11.5 h. Substantial interindividual variability was observed, with concentrations reaching up to 1.5 µg/mL.

Visual inspection of the concentration–time profiles demonstrated that plasma tigecycline concentrations remained above the EUCAST MIC breakpoint for Gram-positive organisms (0.25 µg/mL) during approximately 80% of the dosing interval, whereas concentrations exceeded the Gram-negative breakpoint (0.5 µg/mL) for approximately 40% of the dosing interval, as seen in Fig. 1. These findings suggest that standard dosing regimens may result in subtherapeutic exposure for Gram-negative infections in ECMO patients. Microbiological analysis identified methicillin-resistant Staphylococcus aureus (MRSA) (*n* = 1), Enterococcus faecalis (*n* = 1), Staphylococcus capitis (*n* = 1), and Streptococcus pyogenes (*n* = 1), all with an MIC of 0.25 µg/mL for TGC. Additionally, five patients (33%) had infections caused by multidrug-resistant Gram-negative bacteria (MDRGN) with an MIC of 0.5 µg/mL.

### Comparison of pharmacokinetic parameters by ECMO configuration

Pharmacokinetic parameters were summarized according to ECMO configuration. Patients receiving VA-ECMO showed numerically higher TGC exposure than those receiving VV-ECMO. The median C_max_ was 1.15 µg/mL (IQR: 1.0–1.3) in the VA-ECMO group compared with 0.48 µg/mL (IQR: 0.30–0.60) in the VV-ECMO group. The median AUC was 7.3 µg·h/mL (IQR: 6.8–7.8) in the VA-ECMO group and 2.65 µg·h/mL (IQR: 1.9–3.3) in the VV-ECMO group. Median trough concentrations (C_min_) were also higher in the VA-ECMO group (0.50 µg/mL, IQR: 0.50–0.50) than in the VV-ECMO group (0.10 µg/mL, IQR: 0.09–0.20). Median clearance was lower in the VA-ECMO group (7.0 L/h, IQR: 6.5–7.46) than in the VV-ECMO group (Table 2). Given the very small number of patients receiving VA-ECMO (*n* = 2), these findings should be interpreted as descriptive and exploratory.

**Table 2:** Tigecycline pharmacokinetic parameters between VA- and VV-ECMO

Pharmacokinetic parameter	VV (*n*)	VV median [IQR]	VA (*n*)	VA median [IQR]
C_max_ (ug/mL)	13	0.48 [0.30–0.60]	2	1.15 [1.0–1.3]
C_min_ (ug/mL)	13	0.10 [0.09–0.2]	2	0.5 [0.5–0.5]
AUC_0_12_ (ug.h/mL)	13	2.65 [1.9–3.3]	2	7.3 [6.8–7.8]
Clearance (L/h)	13	18.5 [15.2–26.3]	2	7.0 [6.5–7.5]

*AUC*₀_₁₂ area under the plasma concentration–time curve from 0 to 12 h, *C*_*max*_ maximum plasma concentration, *C*_*min*_ minimum (trough) plasma concentration, *CL* total body clearance, *ECMO* extracorporeal membrane oxygenation, *IQR* interquartile range, *VA* venoarterial, *VV* venovenous.Values are presented as median [interquartile range]

**Fig. 2:**
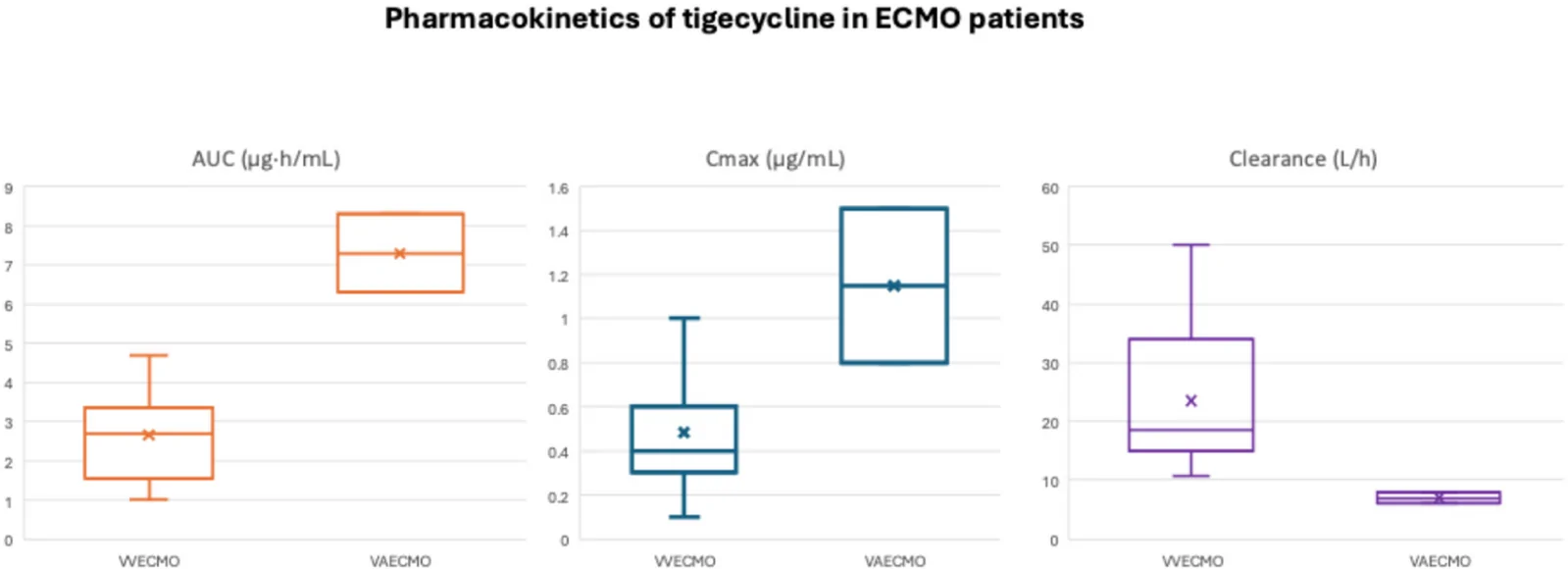
Pharmacokinetics of tigecycline in ECMO patients

Spearman correlation analysis demonstrated a strong positive correlation between C_max_ and AST (ρ = 0.71, *p* = 0.003), and a moderate positive correlation between C_max_ and GGT (ρ = 0.56, *p* = 0.030). Weak to moderate correlations were observed between total bilirubin and TGC pharmacokinetic parameters; however, these associations were not statistically significant. Likewise, no significant correlations were observed between pharmacokinetic parameters and ALT, ALP, or albumin levels (*p* > 0.05) Fig. 2.

## Discussion

In this prospective multicenter pilot study, we characterized the pharmacokinetics of TGC in critically ill patients receiving ECMO support. Our findings demonstrate substantial interindividual variability in TGC plasma concentrations within this cohort. Median peak concentrations were observed shortly after infusion, whereas trough concentrations remained low. Standard dosing resulted in plasma concentrations exceeding EUCAST MIC breakpoints for most Gram-positive pathogens, while target attainment for Gram-negative pathogens was limited. Patients receiving VA-ECMO showed numerically higher TGC exposure than those receiving VV-ECMO. However, these findings should be interpreted with caution given the small number of patients in the VA-ECMO group. In addition, significant positive correlations were observed between C_max_ and both AST and GGT, whereas correlations between total bilirubin and TGC pharmacokinetic parameters did not reach statistical significance. Overall, our findings highlight the considerable pharmacokinetic variability of TGC in critically ill patients receiving ECMO support.

Previous studies investigating TGC pharmacokinetics in critically ill patients have demonstrated substantial interindividual variability, largely attributed to the drug’s high volume of distribution and predominant hepatic elimination [7]. König et al. reported increased TGC exposure in patients with impaired liver function, highlighting hepatic clearance as an important determinant of TGC pharmacokinetics [13]. In our study, significant positive correlations were observed between Cmax and both AST and GGT. Although weak to moderate correlations were also observed between total bilirubin and TGC pharmacokinetic parameters, these associations did not reach statistical significance. Together, these findings suggest that hepatic dysfunction may contribute to interindividual variability in TGC exposure.

In the context of ECMO, published data on TGC pharmacokinetics remain limited. Veinstein et al. described a single patient receiving ECMO whose TGC pharmacokinetic profile was comparable to that reported in critically ill patients without ECMO support, suggesting minimal sequestration within the ECMO circuit [14]. Similarly, Zhang et al. demonstrated minimal TGC adsorption in an ex vivo ECMO circuit model [15]. These findings differ from those reported for several other antimicrobials, including beta-lactams and fluoroquinolones, which may undergo clinically relevant ECMO-related drug loss [2, 16]. In our study, patients receiving VA-ECMO showed numerically higher TGC exposure than those receiving VV-ECMO. However, because only two patients received VA-ECMO, these observations should be considered exploratory and require confirmation in larger studies before any conclusions regarding the effect of ECMO configuration on TGC pharmacokinetics can be drawn.

### Mechanistic explanations

The observed differences in TGC exposure between ECMO configurations may reflect several physiological and circuit-related factors. VA-ECMO provides both cardiac and respiratory support and is associated with altered systemic perfusion and reduced pulsatile blood flow, which may influence tissue drug distribution and contribute to higher circulating plasma concentrations [2]. In contrast, VV-ECMO primarily provides respiratory support while preserving native cardiac output, potentially allowing greater tissue distribution and lower plasma concentrations. Similar differences in drug exposure between VA-ECMO and VV-ECMO have been reported for other antibiotics, including beta-lactams and aminoglycosides [21]. However, given the limited number of VA-ECMO patients included in our study, these proposed mechanisms remain speculative and require confirmation in larger studies.

ECMO circuits may also influence drug pharmacokinetics through drug sequestration within circuit components. However, TGC has relatively low lipophilicity (logP ~ 0.8) and moderate to high plasma protein binding (70–80%), properties that are associated with minimal circuit adsorption. This is supported by the ex vivo study by Zhang et al. and the clinical observations reported by Veinstein et al., both of which demonstrated minimal TGC sequestration within ECMO circuits [14, 15]. Therefore, the pharmacokinetic variability observed in our study is more likely related to patient-specific factors than to drug loss within the ECMO circuit. Critical illness itself, including capillary leak, aggressive fluid resuscitation, and altered organ function, may further contribute to variability in TGC exposure [22, 23].

### Clinical implications

These findings have important clinical implications for the management of infections in patients receiving ECMO support. The substantial interindividual variability in TGC exposure observed in this study, together with the limited attainment of EUCAST MIC breakpoints for Gram-negative pathogens, suggests that standard dosing may not provide adequate exposure in all patients, particularly when treating less susceptible organisms [7, 13]. Although patients receiving VA-ECMO showed numerically higher TGC exposure than those receiving VV-ECMO, this observation should be interpreted with caution because of the very small number of VA-ECMO patients and requires confirmation in larger studies before configuration-specific dosing recommendations can be made. Although TGC efficacy is generally best described by the AUC/MIC ratio, EUCAST MIC breakpoints were used in this pilot study as a descriptive reference for exposure assessment. Because organism-specific MIC values were unavailable for all patients, a formal AUC/MIC target attainment analysis was not feasible. Future studies should evaluate pharmacokinetic target attainment using AUC/MIC-based approaches. Moreover, given the marked variability in TGC exposure, therapeutic drug monitoring, where available, may help individualize dosing and optimize treatment in critically ill patients receiving ECMO [8, 24].

## Limitations

This pilot study has several limitations that should be acknowledged. First, the relatively small sample size, particularly the limited number of patients receiving VA-ECMO, restricts the generalizability of our findings and limits the interpretation of comparisons between ECMO configurations. Consequently, the observed differences between VA-ECMO and VV-ECMO should be considered exploratory. Second, pharmacokinetic analysis was performed using non-compartmental analysis, which was considered appropriate for this exploratory pilot study. Given the limited sample size of this pilot study (particularly only two VA-ECMO patients), construction of a robust population pharmacokinetic model was not feasible. Therefore, non-compartmental analysis was selected as an exploratory descriptive approach. Finally, therapeutic drug monitoring was not available in real time during the study period, limiting the opportunity to individualize dosing based on measured drug concentrations.

## Conclusion

This prospective pilot study provides preliminary insights into TGC pharmacokinetics in critically ill patients receiving ECMO support, demonstrating substantial interindividual variability in drug exposure. Standard dosing resulted in plasma concentrations exceeding EUCAST MIC breakpoints for most Gram-positive pathogens, whereas target attainment for Gram-negative pathogens was more limited. Patients receiving VA-ECMO showed numerically higher TGC exposure than those receiving VV-ECMO. However, these findings should be interpreted with caution because of the small number of VA-ECMO patients. Further studies with larger patient cohorts are needed to confirm these observations and to evaluate the potential role of therapeutic drug monitoring and individualized dosing strategies for optimizing TGC therapy in this complex patient population.

## Data Availability

The datasets generated and/or analysed during the current study are available from the corresponding author on reasonable request.

## Abbreviations

ALP: Alkaline phosphatase
ALT: Alanine aminotransferase
AST: Aspartate aminotransferase
AUC₀–₁₂: Area under the concentration-time curve over 12 h
CL: Clearance
Cmax: Maximum plasma concentration
Cmin: Minimum plasma concentration
ECMO: Extracorporeal membrane oxygenation
GGT: Gamma-glutamyl transferase
ICU: Intensive care unit
IQR: Interquartile range
LC-MS/MS: Liquid chromatography–tandem mass spectrometry
MIC: Minimum inhibitory concentration
TGC: Tigecycline
VA: Venoarterial
VV: Venovenous
